# Poly[diaqua­tris­(μ_4_-1,3-phenyl­ene­diacetato)­dicerium(III)]

**DOI:** 10.1107/S1600536811003801

**Published:** 2011-02-09

**Authors:** Zhu-Qing Gao, Hong-Jin Li, Jin-Zhong Gu

**Affiliations:** a School of Chemistry and Biology Engineering, Taiyuan University of Science and Technology, Taiyuan 030021, People’s Republic of China; b Key Laboratory of Nonferrous Metal Chemistry and Resources Utilization of Gansu Province, College of Chemistry and Chemical Engineering, Lanzhou University, Lanzhou 730000, People’s Republic of China

## Abstract

In the title coordination polymer, [Ce_2_(C_10_H_8_O_4_)_3_(H_2_O)_2_]_*n*_, each Ce^III^ atom is nine-coordinated by eight O atoms from six different 1,3-phenyl­enediacetate (pda) bivalent anions and one O atom from a coordinated water mol­ecule, forming a distorted tricapped trigonal–prismatic coordination geometry. Eight Ce^III^ ions and twelve pda ligands form a large [Ce_8_(pda)_12_] ring, and four Ce^III^ ions and six pda ligands form a small [Ce_4_(pda)_6_] ring. The rings are further connected by the coordination inter­actions of pda ligands and Ce^III^, generating a three-dimensional supra­molecular framework.

## Related literature

For the structures and properties of lanthanide coordination compounds, see: Chen *et al.* (2008 ▶); Lv *et al.* (2010 ▶). For bond lengths and angles in other complexes with nine-coordinate Ce^III^, see: Chen *et al.* (2008 ▶); Ramya *et al.* (2010 ▶). 
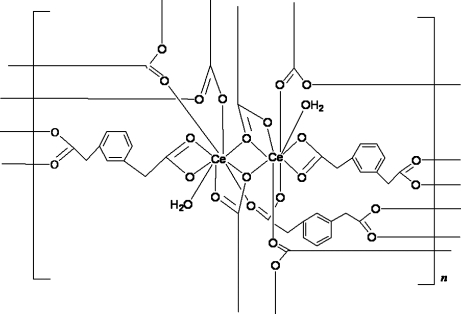


## Experimental

### 

#### Crystal data

[Ce_2_(C_10_H_8_O_4_)_3_(H_2_O)_2_]
*M* *_r_* = 892.76
Triclinic, 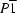

*a* = 10.5552 (2) Å
*b* = 12.0275 (2) Å
*c* = 12.4612 (2) Å
α = 105.686 (1)°
β = 96.748 (1)°
γ = 92.949 (1)°
*V* = 1506.77 (5) Å^3^
*Z* = 2
Mo *K*α radiation
μ = 3.06 mm^−1^
*T* = 296 K
0.25 × 0.23 × 0.20 mm

#### Data collection

Bruker SMART CCD area-detector diffractometer
Absorption correction: multi-scan (*SADABS*; Bruker, 1997 ▶) *T*_min_ = 0.516, *T*_max_ = 0.580
8224 measured reflections
5545 independent reflections
4673 reflections with *I* > 2σ(*I*)
*R*_int_ = 0.018

#### Refinement

*R*[*F* ^2^ > 2σ(*F* ^2^)] = 0.025
*wR*(*F* ^2^) = 0.054
*S* = 1.01
5545 reflections
431 parameters
6 restraints
H atoms treated by a mixture of independent and constrained refinement
Δρ_max_ = 0.53 e Å^−3^
Δρ_min_ = −0.38 e Å^−3^

### 

Data collection: *SMART* (Bruker, 1997 ▶); cell refinement: *SAINT* (Bruker, 1997 ▶); data reduction: *SAINT*; program(s) used to solve structure: *SHELXS97* (Sheldrick, 2008 ▶); program(s) used to refine structure: *SHELXL97* (Sheldrick, 2008 ▶); molecular graphics: *SHELXTL* (Sheldrick, 2008 ▶); software used to prepare material for publication: *SHELXTL*.

## Supplementary Material

Crystal structure: contains datablocks I, global. DOI: 10.1107/S1600536811003801/pv2385sup1.cif

Structure factors: contains datablocks I. DOI: 10.1107/S1600536811003801/pv2385Isup2.hkl

Additional supplementary materials:  crystallographic information; 3D view; checkCIF report

## Figures and Tables

**Table 1:** Hydrogen-bond geometry (Å, °)

*D*—H⋯*A*	*D*—H	H⋯*A*	*D*⋯*A*	*D*—H⋯*A*
O13—H1*W*⋯O6^i^	0.86 (1)	2.03 (2)	2.860 (4)	161 (4)
O14—H4*W*⋯O1^ii^	0.87 (4)	2.23 (2)	3.051 (4)	159 (5)
O14—H3*W*⋯O9^iii^	0.86 (4)	2.62 (4)	3.180 (4)	123 (4)
O14—H3*W*⋯O1^iv^	0.86 (4)	2.07 (5)	2.873 (4)	155 (5)
O13—H2*W*⋯O9	0.86 (5)	2.81 (7)	3.037 (4)	97 (5)
O13—H2*W*⋯O1^ii^	0.86 (5)	2.75 (5)	2.898 (4)	91 (3)

Symmetry codes: (i) 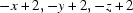; (ii) 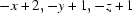; (iii) 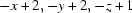; (iv) 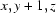.

**Fig. 1.**
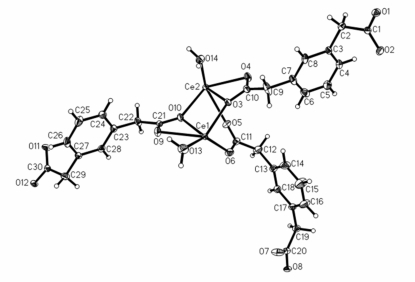
A drawing of the asymmetric unit of the title complex, showing the atom-labeling scheme. Displacement ellipsoids are drawn at the 30% probability level.

**Fig. 2.**
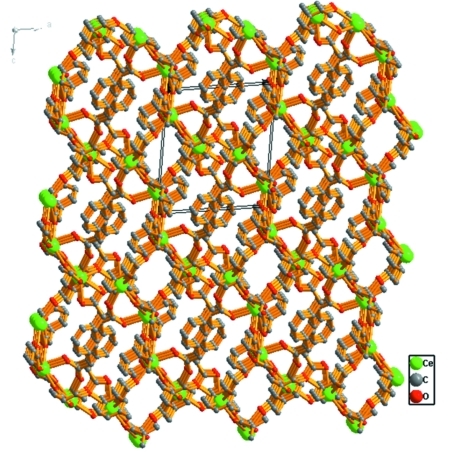
Unit cell packing of the title complex showing three dimensional framework formed by a large [Ce8(pda)12] and a small [Ce4(pda)6] ring.

## supplementary crystallographic information

### Comment

The lanthanide coordination polymers have shown not only versatile architectures but also desirable properties, e.g., luminescent, magnetic, catalytic, and gas absorption and separation properties (Chen *et al.*, 2008; Lv *et al.*, 2010). In order to extend the investigation in this field, we have designed and synthesized the title lanthanide coordination polymer by choosing 1,3-phenylendiacetic acid as a functional ligand, and report its crystal structure in this paper.

The asymmetric unit of the title complex (Fig. 1) contains two crystallographically unique Ce^III^ ions, three 1,3-phenylenediacetate (pda) ligands, and two coordinated water molecules. Both Ce1 and Ce2 are nine-coordinated with a distorted tricapped trigonal-prismatic geometry; the nine coordination sites are occupied by one oxygen atom from one coordinated water molecule and eight O atoms from six different pda ligands.

The Ce—O bond distances in the title complex are in the range 2.445 (2)–2.764 (3) Å, which are comparable to those reported for other Ce—O complexes (Chen *et al.*, 2008; Ramya *et al.*, 2010). The pda ligands adopt two coordination modes of *µ*_4_-hexadentate and *µ*_4_-pentadentate. Eight Ce^III^ ions and twelve pda ligands form a large [Ce_8_(pda)_12_] ring, and four Ce^III^ ions and six pda ligands form a small [Ce_4_(pda)_6_] ring (Fig. 2). The rings are further connected by the coordination interactions of pda ligands and Ce^III^ to generate a three-dimensional supramolecular framework (Fig. 2).

### Experimental

To a solution of cerium nitrate hexahydrate (0.087 g, 0.2 mmol) in water (5 ml) was added an aqueous solution (5 ml) of the ligand (0.058 g, 0.3 mmol) and a drop of triethylamine. The reactants were sealed in a 25-ml Teflon-lined, stainless-steel Parr bomb. The bomb was heated at 433 K for 3 days. Upon cooling, the solution yielded single crystals of the title complex in *ca* 70% yield.

### Refinement

The coordinated water H atoms were located from a different Fourier map and refined with distance constraints O–H = 0.83 (3) Å. The carbon-bound H atoms were placed in geometrically idealized positions, with C–H = 0.93 and 0.97 Å for aryl and methylene H-atoms, respectively, and constrained to ride on their respective parent atoms, with *U*_iso_(H) = 1.2*U*_eq_(C).

### Crystal data

**Table:**

[Ce_2_(C_10_H_8_O_4_)_3_(H_2_O)_2_]	*Z* = 2
*M**_r_* = 892.76	*F*(000) = 872
Triclinic, *P*1	*D*_x_ = 1.968 Mg m^−^^3^
Hall symbol: -P 1	Mo *K*α radiation, λ = 0.71073 Å
*a* = 10.5552 (2) Å	Cell parameters from 3826 reflections
*b* = 12.0275 (2) Å	θ = 2.8–28.1°
*c* = 12.4612 (2) Å	µ = 3.06 mm^−^^1^
α = 105.686 (1)°	*T* = 296 K
β = 96.748 (1)°	Block, colorless
γ = 92.949 (1)°	0.25 × 0.23 × 0.20 mm
*V* = 1506.77 (5) Å^3^	

### Data collection

**Table:**

Bruker SMART CCD area-detector diffractometer	5545 independent reflections
Radiation source: fine-focus sealed tube	4673 reflections with *I* > 2σ(*I*)
graphite	*R*_int_ = 0.018
φ and ω scans	θ_max_ = 25.5°, θ_min_ = 1.8°
Absorption correction: multi-scan (*SADABS*; Bruker, 1997)	*h* = −12→10
*T*_min_ = 0.516, *T*_max_ = 0.580	*k* = −12→14
8224 measured reflections	*l* = −15→14

### Refinement

**Table:**

Refinement on *F*^2^	Primary atom site location: structure-invariant direct methods
Least-squares matrix: full	Secondary atom site location: difference Fourier map
*R*[*F*^2^ > 2σ(*F*^2^)] = 0.025	Hydrogen site location: inferred from neighbouring sites
*wR*(*F*^2^) = 0.054	H atoms treated by a mixture of independent and constrained refinement
*S* = 1.01	*w* = 1/[σ^2^(*F*_o_^2^) + (0.0178*P*)^2^ + 1.4251*P*] where *P* = (*F*_o_^2^ + 2*F*_c_^2^)/3
5545 reflections	(Δ/σ)_max_ = 0.001
431 parameters	Δρ_max_ = 0.53 e Å^−^^3^
6 restraints	Δρ_min_ = −0.38 e Å^−^^3^

### Special details

**Table:**

Experimental. Anal. Calcd for C_30_H_28_Ce_2_O_14_: C, 40.36; H, 3.16. Found: C, 40.68; H, 3.45.
Geometry. All e.s.d.'s (except the e.s.d. in the dihedral angle between two l.s. planes) are estimated using the full covariance matrix. The cell e.s.d.'s are taken into account individually in the estimation of e.s.d.'s in distances, angles and torsion angles; correlations between e.s.d.'s in cell parameters are only used when they are defined by crystal symmetry. An approximate (isotropic) treatment of cell e.s.d.'s is used for estimating e.s.d.'s involving l.s. planes.
Refinement. Refinement of *F*^2^ against ALL reflections. The weighted *R*-factor *wR* and goodness of fit *S* are based on *F*^2^, conventional *R*-factors *R* are based on *F*, with *F* set to zero for negative *F*^2^. The threshold expression of *F*^2^ > σ(*F*^2^) is used only for calculating *R*-factors(gt) *etc*. and is not relevant to the choice of reflections for refinement. *R*-factors based on *F*^2^ are statistically about twice as large as those based on *F*, and *R*- factors based on ALL data will be even larger.

### Fractional atomic coordinates and isotropic or equivalent isotropic displacement parameters (Å^2^)

**Table:**

	*x*	*y*	*z*	*U*_iso_*/*U*_eq_	
Ce1	0.969885 (19)	0.972983 (17)	0.817505 (16)	0.01890 (6)	
Ce2	0.693423 (19)	0.963262 (18)	0.545875 (17)	0.02092 (6)	
C1	0.8215 (4)	0.1335 (3)	0.3896 (3)	0.0252 (9)	
C2	0.7819 (4)	0.2362 (3)	0.3509 (3)	0.0304 (9)	
H2A	0.7153	0.2093	0.2869	0.036*	
H2B	0.8547	0.2688	0.3253	0.036*	
C3	0.7333 (4)	0.3315 (3)	0.4388 (3)	0.0252 (9)	
C4	0.6200 (4)	0.3156 (3)	0.4807 (4)	0.0323 (10)	
H4	0.5704	0.2452	0.4533	0.039*	
C5	0.5802 (4)	0.4039 (4)	0.5630 (4)	0.0382 (11)	
H5	0.5045	0.3925	0.5915	0.046*	
C6	0.6527 (4)	0.5085 (4)	0.6023 (4)	0.0420 (11)	
H6	0.6257	0.5673	0.6580	0.050*	
C7	0.7643 (4)	0.5279 (3)	0.5609 (4)	0.0343 (10)	
C8	0.8041 (4)	0.4383 (3)	0.4799 (4)	0.0308 (9)	
H8	0.8803	0.4500	0.4523	0.037*	
C9	0.8450 (4)	0.6422 (4)	0.6046 (5)	0.0529 (14)	
H9A	0.8711	0.6550	0.6846	0.063*	
H9B	0.9222	0.6352	0.5689	0.063*	
C10	0.7854 (4)	0.7485 (3)	0.5882 (3)	0.0276 (9)	
C11	0.6407 (4)	0.9107 (4)	0.8043 (3)	0.0297 (9)	
C12	0.5345 (4)	0.8419 (4)	0.8368 (4)	0.0418 (11)	
H12A	0.4669	0.8925	0.8557	0.050*	
H12B	0.4994	0.7798	0.7706	0.050*	
C13	0.5660 (4)	0.7886 (4)	0.9325 (3)	0.0326 (10)	
C14	0.6567 (5)	0.7105 (4)	0.9286 (4)	0.0480 (13)	
H14	0.7021	0.6910	0.8675	0.058*	
C15	0.6803 (6)	0.6612 (5)	1.0154 (5)	0.0671 (17)	
H15	0.7429	0.6093	1.0131	0.081*	
C16	0.6130 (5)	0.6871 (5)	1.1058 (4)	0.0553 (14)	
H16	0.6291	0.6509	1.1625	0.066*	
C17	0.5223 (4)	0.7657 (4)	1.1136 (3)	0.0310 (9)	
C18	0.4988 (4)	0.8160 (4)	1.0256 (3)	0.0313 (9)	
H18	0.4371	0.8688	1.0286	0.038*	
C19	0.4520 (4)	0.7942 (4)	1.2146 (3)	0.0329 (10)	
H19A	0.4176	0.7223	1.2255	0.040*	
H19B	0.3802	0.8372	1.1991	0.040*	
C20	0.5324 (4)	0.8637 (4)	1.3227 (3)	0.0295 (9)	
C21	0.9293 (4)	1.1831 (3)	0.7398 (3)	0.0243 (8)	
C22	0.8979 (4)	1.2973 (3)	0.7189 (4)	0.0355 (10)	
H22A	0.8243	1.3228	0.7557	0.043*	
H22B	0.8735	1.2846	0.6387	0.043*	
C23	1.0052 (4)	1.3934 (3)	0.7592 (3)	0.0282 (9)	
C24	1.1166 (4)	1.3857 (4)	0.7107 (4)	0.0380 (11)	
H24	1.1258	1.3210	0.6521	0.046*	
C25	1.2141 (5)	1.4732 (4)	0.7485 (4)	0.0431 (12)	
H25	1.2887	1.4673	0.7151	0.052*	
C26	1.2020 (4)	1.5698 (4)	0.8358 (4)	0.0380 (11)	
H26	1.2692	1.6277	0.8615	0.046*	
C27	1.0919 (4)	1.5808 (3)	0.8847 (3)	0.0284 (9)	
C28	0.9938 (4)	1.4923 (3)	0.8464 (3)	0.0307 (9)	
H28	0.9189	1.4990	0.8793	0.037*	
C29	1.0769 (5)	1.6894 (3)	0.9753 (3)	0.0395 (11)	
H29A	1.1572	1.7125	1.0253	0.047*	
H29B	1.0124	1.6723	1.0193	0.047*	
C30	1.0393 (4)	1.7894 (3)	0.9306 (3)	0.0260 (9)	
H1W	1.231 (4)	1.050 (4)	0.9665 (10)	0.056 (16)*	
H2W	1.245 (5)	1.061 (5)	0.856 (3)	0.12 (3)*	
H3W	0.905 (4)	0.933 (4)	0.408 (3)	0.059 (16)*	
H4W	0.965 (3)	0.898 (5)	0.502 (4)	0.11 (3)*	
O1	0.9012 (3)	0.0713 (2)	0.3390 (2)	0.0325 (7)	
O2	0.7753 (3)	0.1133 (2)	0.4708 (3)	0.0439 (8)	
O3	0.8387 (2)	0.8457 (2)	0.6504 (2)	0.0283 (6)	
O4	0.6920 (3)	0.7436 (2)	0.5162 (2)	0.0348 (7)	
O5	0.6106 (3)	0.9377 (2)	0.7151 (2)	0.0345 (7)	
O6	0.7483 (3)	0.9369 (3)	0.8664 (2)	0.0370 (7)	
O7	0.6493 (3)	0.8820 (3)	1.3289 (2)	0.0519 (9)	
O8	0.4748 (2)	0.8997 (2)	1.4094 (2)	0.0289 (6)	
O9	1.0327 (3)	1.1711 (2)	0.7907 (2)	0.0337 (7)	
O10	0.8427 (3)	1.0968 (2)	0.7048 (2)	0.0312 (6)	
O11	1.0126 (3)	1.7751 (2)	0.8285 (2)	0.0411 (8)	
O12	1.0372 (3)	1.8880 (2)	0.9995 (2)	0.0336 (7)	
O13	1.2073 (3)	1.0241 (3)	0.8950 (3)	0.0448 (8)	
O14	0.8946 (3)	0.8976 (3)	0.4580 (3)	0.0353 (7)	

### Atomic displacement parameters (Å^2^)

**Table:**

	*U*^11^	*U*^22^	*U*^33^	*U*^12^	*U*^13^	*U*^23^
Ce1	0.02518 (12)	0.01494 (11)	0.01602 (11)	0.00166 (9)	0.00181 (9)	0.00381 (8)
Ce2	0.02308 (12)	0.02296 (13)	0.01748 (11)	0.00208 (9)	0.00230 (9)	0.00709 (9)
C1	0.027 (2)	0.023 (2)	0.024 (2)	−0.0039 (17)	0.0053 (17)	0.0038 (16)
C2	0.038 (2)	0.031 (2)	0.025 (2)	0.0049 (19)	0.0041 (18)	0.0119 (18)
C3	0.031 (2)	0.020 (2)	0.025 (2)	0.0041 (17)	−0.0026 (17)	0.0075 (16)
C4	0.033 (2)	0.022 (2)	0.042 (3)	0.0004 (18)	0.0025 (19)	0.0112 (19)
C5	0.036 (2)	0.036 (3)	0.046 (3)	0.007 (2)	0.014 (2)	0.014 (2)
C6	0.050 (3)	0.031 (3)	0.041 (3)	0.015 (2)	0.005 (2)	0.002 (2)
C7	0.034 (2)	0.021 (2)	0.043 (3)	0.0067 (18)	−0.010 (2)	0.0059 (19)
C8	0.026 (2)	0.023 (2)	0.046 (3)	0.0031 (17)	0.0003 (19)	0.0162 (19)
C9	0.044 (3)	0.022 (2)	0.079 (4)	0.003 (2)	−0.021 (3)	0.003 (2)
C10	0.028 (2)	0.022 (2)	0.030 (2)	0.0006 (17)	0.0058 (18)	0.0021 (17)
C11	0.030 (2)	0.034 (2)	0.029 (2)	0.0025 (18)	0.0098 (18)	0.0119 (19)
C12	0.035 (2)	0.058 (3)	0.032 (2)	−0.012 (2)	−0.001 (2)	0.017 (2)
C13	0.032 (2)	0.039 (3)	0.025 (2)	−0.007 (2)	−0.0004 (18)	0.0096 (19)
C14	0.061 (3)	0.055 (3)	0.032 (3)	0.018 (3)	0.021 (2)	0.009 (2)
C15	0.092 (4)	0.070 (4)	0.061 (4)	0.050 (3)	0.043 (3)	0.032 (3)
C16	0.081 (4)	0.062 (4)	0.037 (3)	0.031 (3)	0.023 (3)	0.027 (3)
C17	0.037 (2)	0.035 (2)	0.021 (2)	0.0040 (19)	0.0017 (18)	0.0074 (18)
C18	0.030 (2)	0.032 (2)	0.031 (2)	−0.0026 (18)	0.0007 (18)	0.0094 (18)
C19	0.031 (2)	0.041 (3)	0.027 (2)	−0.0002 (19)	0.0084 (18)	0.0084 (19)
C20	0.032 (2)	0.036 (2)	0.021 (2)	0.0065 (19)	0.0037 (17)	0.0073 (18)
C21	0.035 (2)	0.017 (2)	0.020 (2)	0.0015 (17)	0.0055 (17)	0.0031 (16)
C22	0.048 (3)	0.018 (2)	0.036 (3)	0.0030 (19)	−0.006 (2)	0.0066 (18)
C23	0.040 (2)	0.019 (2)	0.027 (2)	0.0046 (18)	0.0004 (18)	0.0106 (17)
C24	0.063 (3)	0.021 (2)	0.032 (2)	0.008 (2)	0.016 (2)	0.0064 (18)
C25	0.049 (3)	0.034 (3)	0.057 (3)	0.009 (2)	0.022 (2)	0.023 (2)
C26	0.043 (3)	0.024 (2)	0.049 (3)	−0.0022 (19)	−0.001 (2)	0.017 (2)
C27	0.049 (3)	0.015 (2)	0.021 (2)	0.0047 (18)	−0.0012 (18)	0.0079 (16)
C28	0.046 (3)	0.023 (2)	0.027 (2)	0.0088 (19)	0.0104 (19)	0.0114 (17)
C29	0.068 (3)	0.025 (2)	0.025 (2)	0.008 (2)	0.001 (2)	0.0085 (18)
C30	0.033 (2)	0.018 (2)	0.026 (2)	0.0004 (17)	0.0030 (17)	0.0054 (17)
O1	0.0348 (16)	0.0353 (17)	0.0328 (16)	0.0105 (13)	0.0152 (13)	0.0126 (13)
O2	0.069 (2)	0.0309 (17)	0.0450 (19)	0.0187 (15)	0.0341 (17)	0.0200 (14)
O3	0.0362 (16)	0.0195 (14)	0.0251 (15)	−0.0021 (12)	−0.0011 (12)	0.0024 (12)
O4	0.0334 (16)	0.0261 (16)	0.0386 (17)	−0.0045 (13)	−0.0105 (14)	0.0061 (13)
O5	0.0346 (16)	0.0446 (18)	0.0312 (16)	0.0050 (14)	0.0096 (13)	0.0195 (14)
O6	0.0315 (16)	0.0486 (19)	0.0291 (16)	−0.0073 (14)	0.0031 (13)	0.0106 (14)
O7	0.0293 (18)	0.090 (3)	0.0269 (17)	−0.0011 (17)	0.0046 (13)	0.0015 (17)
O8	0.0347 (16)	0.0355 (16)	0.0174 (14)	0.0093 (13)	0.0063 (12)	0.0065 (12)
O9	0.0329 (16)	0.0245 (15)	0.0441 (18)	−0.0023 (13)	−0.0017 (14)	0.0139 (13)
O10	0.0388 (16)	0.0208 (15)	0.0312 (16)	−0.0048 (12)	−0.0050 (13)	0.0077 (12)
O11	0.077 (2)	0.0238 (16)	0.0200 (16)	0.0086 (15)	−0.0031 (15)	0.0050 (12)
O12	0.0596 (19)	0.0134 (14)	0.0252 (15)	0.0051 (13)	0.0078 (14)	−0.0002 (12)
O13	0.0350 (18)	0.056 (2)	0.038 (2)	−0.0053 (16)	−0.0049 (16)	0.0100 (18)
O14	0.0346 (17)	0.0395 (19)	0.0366 (18)	0.0087 (14)	0.0080 (15)	0.0165 (15)

### Geometric parameters (Å, °)

**Table:**

Ce1—O3	2.445 (2)	C14—C15	1.371 (7)
Ce1—O12^i^	2.448 (2)	C14—H14	0.9300
Ce1—O1^ii^	2.465 (3)	C15—C16	1.376 (6)
Ce1—O11^iii^	2.482 (3)	C15—H15	0.9300
Ce1—O6	2.531 (3)	C16—C17	1.372 (6)
Ce1—O13	2.558 (3)	C16—H16	0.9300
Ce1—O9	2.559 (3)	C17—C18	1.393 (5)
Ce1—O10	2.624 (3)	C17—C19	1.505 (5)
Ce1—O12^iii^	2.764 (3)	C18—H18	0.9300
Ce1—C30^iii^	3.001 (4)	C19—C20	1.511 (5)
Ce2—O2^iv^	2.412 (3)	C19—H19A	0.9700
Ce2—O5	2.462 (3)	C19—H19B	0.9700
Ce2—O8^v^	2.494 (3)	C20—O7	1.232 (5)
Ce2—O10	2.504 (3)	C20—O8	1.285 (4)
Ce2—O4	2.565 (3)	C20—Ce2^vii^	2.989 (4)
Ce2—O14	2.566 (3)	C21—O9	1.231 (4)
Ce2—O7^vi^	2.590 (3)	C21—O10	1.291 (4)
Ce2—O3	2.606 (3)	C21—C22	1.512 (5)
Ce2—O8^vi^	2.645 (3)	C22—C23	1.513 (5)
Ce2—C20^vi^	2.989 (4)	C22—H22A	0.9700
C1—O2	1.248 (4)	C22—H22B	0.9700
C1—O1	1.264 (4)	C23—C24	1.380 (6)
C1—C2	1.504 (5)	C23—C28	1.398 (5)
C2—C3	1.514 (5)	C24—C25	1.377 (6)
C2—H2A	0.9700	C24—H24	0.9300
C2—H2B	0.9700	C25—C26	1.383 (6)
C3—C4	1.384 (5)	C25—H25	0.9300
C3—C8	1.388 (5)	C26—C27	1.371 (6)
C4—C5	1.384 (6)	C26—H26	0.9300
C4—H4	0.9300	C27—C28	1.389 (5)
C5—C6	1.374 (6)	C27—C29	1.507 (5)
C5—H5	0.9300	C28—H28	0.9300
C6—C7	1.375 (6)	C29—C30	1.507 (5)
C6—H6	0.9300	C29—H29A	0.9700
C7—C8	1.383 (6)	C29—H29B	0.9700
C7—C9	1.510 (6)	C30—O11	1.233 (4)
C8—H8	0.9300	C30—O12	1.266 (4)
C9—C10	1.505 (6)	C30—Ce1^iv^	3.001 (4)
C9—H9A	0.9700	O1—Ce1^ii^	2.465 (3)
C9—H9B	0.9700	O2—Ce2^iii^	2.412 (3)
C10—O4	1.241 (4)	O7—Ce2^vii^	2.590 (3)
C10—O3	1.275 (4)	O8—Ce2^v^	2.494 (3)
C11—O5	1.252 (5)	O8—Ce2^vii^	2.645 (3)
C11—O6	1.269 (5)	O11—Ce1^iv^	2.482 (3)
C11—C12	1.516 (5)	O12—Ce1^i^	2.448 (2)
C12—C13	1.511 (6)	O12—Ce1^iv^	2.764 (3)
C12—H12A	0.9700	O13—H1W	0.864 (10)
C12—H12B	0.9700	O13—H2W	0.86 (5)
C13—C14	1.372 (6)	O14—H3W	0.86 (4)
C13—C18	1.403 (6)	O14—H4W	0.87 (4)
			
O3—Ce1—O12^i^	144.20 (9)	C7—C8—H8	119.1
O3—Ce1—O1^ii^	70.91 (9)	C3—C8—H8	119.1
O12^i^—Ce1—O1^ii^	141.62 (10)	C10—C9—C7	117.4 (4)
O3—Ce1—O11^iii^	76.19 (9)	C10—C9—H9A	107.9
O12^i^—Ce1—O11^iii^	113.90 (9)	C7—C9—H9A	107.9
O1^ii^—Ce1—O11^iii^	84.02 (9)	C10—C9—H9B	107.9
O3—Ce1—O6	71.59 (9)	C7—C9—H9B	107.9
O12^i^—Ce1—O6	74.44 (9)	H9A—C9—H9B	107.2
O1^ii^—Ce1—O6	142.49 (9)	O4—C10—O3	120.9 (4)
O11^iii^—Ce1—O6	88.17 (10)	O4—C10—C9	122.7 (4)
O3—Ce1—O13	138.23 (10)	O3—C10—C9	116.3 (4)
O12^i^—Ce1—O13	77.54 (11)	O5—C11—O6	125.7 (4)
O1^ii^—Ce1—O13	70.46 (10)	O5—C11—C12	114.0 (4)
O11^iii^—Ce1—O13	84.60 (11)	O6—C11—C12	120.2 (4)
O6—Ce1—O13	145.16 (10)	C13—C12—C11	118.5 (4)
O3—Ce1—O9	111.91 (9)	C13—C12—H12A	107.7
O12^i^—Ce1—O9	74.97 (9)	C11—C12—H12A	107.7
O1^ii^—Ce1—O9	75.76 (9)	C13—C12—H12B	107.7
O11^iii^—Ce1—O9	153.60 (10)	C11—C12—H12B	107.7
O6—Ce1—O9	118.18 (9)	H12A—C12—H12B	107.1
O13—Ce1—O9	72.83 (10)	C14—C13—C18	118.9 (4)
O3—Ce1—O10	69.88 (8)	C14—C13—C12	121.2 (4)
O12^i^—Ce1—O10	93.84 (9)	C18—C13—C12	119.9 (4)
O1^ii^—Ce1—O10	85.74 (9)	C15—C14—C13	119.7 (4)
O11^iii^—Ce1—O10	146.06 (9)	C15—C14—H14	120.2
O6—Ce1—O10	80.57 (9)	C13—C14—H14	120.2
O13—Ce1—O10	121.88 (10)	C14—C15—C16	121.2 (5)
O9—Ce1—O10	49.78 (8)	C14—C15—H15	119.4
O3—Ce1—O12^iii^	118.78 (8)	C16—C15—H15	119.4
O12^i^—Ce1—O12^iii^	65.74 (9)	C17—C16—C15	121.0 (4)
O1^ii^—Ce1—O12^iii^	118.34 (9)	C17—C16—H16	119.5
O11^iii^—Ce1—O12^iii^	48.57 (8)	C15—C16—H16	119.5
O6—Ce1—O12^iii^	81.25 (9)	C16—C17—C18	117.7 (4)
O13—Ce1—O12^iii^	68.50 (10)	C16—C17—C19	119.8 (4)
O9—Ce1—O12^iii^	129.26 (8)	C18—C17—C19	122.5 (4)
O10—Ce1—O12^iii^	155.72 (8)	C17—C18—C13	121.5 (4)
O3—Ce1—C30^iii^	97.22 (9)	C17—C18—H18	119.2
O12^i^—Ce1—C30^iii^	90.49 (10)	C13—C18—H18	119.2
O1^ii^—Ce1—C30^iii^	101.08 (10)	C17—C19—C20	114.7 (3)
O11^iii^—Ce1—C30^iii^	23.65 (9)	C17—C19—H19A	108.6
O6—Ce1—C30^iii^	84.57 (10)	C20—C19—H19A	108.6
O13—Ce1—C30^iii^	75.33 (11)	C17—C19—H19B	108.6
O9—Ce1—C30^iii^	147.13 (10)	C20—C19—H19B	108.6
O10—Ce1—C30^iii^	162.79 (9)	H19A—C19—H19B	107.6
O12^iii^—Ce1—C30^iii^	24.93 (8)	O7—C20—O8	121.0 (4)
O2^iv^—Ce2—O5	140.83 (10)	O7—C20—C19	121.5 (4)
O2^iv^—Ce2—O8^v^	81.37 (9)	O8—C20—C19	117.4 (3)
O5—Ce2—O8^v^	72.26 (9)	O7—C20—Ce2^vii^	59.5 (2)
O2^iv^—Ce2—O10	74.63 (10)	O8—C20—Ce2^vii^	62.15 (19)
O5—Ce2—O10	76.26 (9)	C19—C20—Ce2^vii^	170.4 (3)
O8^v^—Ce2—O10	88.60 (9)	O9—C21—O10	119.7 (3)
O2^iv^—Ce2—O4	140.36 (9)	O9—C21—C22	122.2 (3)
O5—Ce2—O4	77.50 (9)	O10—C21—C22	118.0 (3)
O8^v^—Ce2—O4	132.08 (9)	C21—C22—C23	115.1 (3)
O10—Ce2—O4	119.23 (8)	C21—C22—H22A	108.5
O2^iv^—Ce2—O14	71.63 (10)	C23—C22—H22A	108.5
O5—Ce2—O14	131.71 (10)	C21—C22—H22B	108.5
O8^v^—Ce2—O14	152.91 (9)	C23—C22—H22B	108.5
O10—Ce2—O14	86.39 (10)	H22A—C22—H22B	107.5
O4—Ce2—O14	72.51 (9)	C24—C23—C28	118.4 (4)
O2^iv^—Ce2—O7^vi^	73.78 (11)	C24—C23—C22	120.8 (4)
O5—Ce2—O7^vi^	139.87 (10)	C28—C23—C22	120.8 (4)
O8^v^—Ce2—O7^vi^	103.64 (9)	C25—C24—C23	120.4 (4)
O10—Ce2—O7^vi^	143.78 (10)	C25—C24—H24	119.8
O4—Ce2—O7^vi^	77.21 (11)	C23—C24—H24	119.8
O14—Ce2—O7^vi^	67.16 (10)	C24—C25—C26	120.5 (4)
O2^iv^—Ce2—O3	123.59 (10)	C24—C25—H25	119.7
O5—Ce2—O3	67.37 (9)	C26—C25—H25	119.7
O8^v^—Ce2—O3	137.36 (8)	C27—C26—C25	120.5 (4)
O10—Ce2—O3	69.30 (8)	C27—C26—H26	119.7
O4—Ce2—O3	50.05 (8)	C25—C26—H26	119.7
O14—Ce2—O3	64.34 (9)	C26—C27—C28	118.8 (4)
O7^vi^—Ce2—O3	115.83 (10)	C26—C27—C29	120.0 (4)
O2^iv^—Ce2—O8^vi^	98.83 (10)	C28—C27—C29	121.1 (4)
O5—Ce2—O8^vi^	96.30 (8)	C27—C28—C23	121.4 (4)
O8^v^—Ce2—O8^vi^	66.00 (10)	C27—C28—H28	119.3
O10—Ce2—O8^vi^	154.56 (8)	C23—C28—H28	119.3
O4—Ce2—O8^vi^	81.72 (8)	C27—C29—C30	113.7 (3)
O14—Ce2—O8^vi^	115.35 (9)	C27—C29—H29A	108.8
O7^vi^—Ce2—O8^vi^	49.47 (8)	C30—C29—H29A	108.8
O3—Ce2—O8^vi^	130.76 (8)	C27—C29—H29B	108.8
O2^iv^—Ce2—C20^vi^	87.36 (11)	C30—C29—H29B	108.8
O5—Ce2—C20^vi^	118.44 (10)	H29A—C29—H29B	107.7
O8^v^—Ce2—C20^vi^	86.33 (10)	O11—C30—O12	120.8 (4)
O10—Ce2—C20^vi^	161.83 (10)	O11—C30—C29	120.3 (3)
O4—Ce2—C20^vi^	76.41 (10)	O12—C30—C29	118.9 (3)
O14—Ce2—C20^vi^	90.21 (11)	O11—C30—Ce1^iv^	53.8 (2)
O7^vi^—Ce2—C20^vi^	24.18 (9)	O12—C30—Ce1^iv^	67.0 (2)
O3—Ce2—C20^vi^	124.77 (10)	C29—C30—Ce1^iv^	173.9 (3)
O8^vi^—Ce2—C20^vi^	25.44 (9)	C1—O1—Ce1^ii^	148.5 (3)
O2—C1—O1	122.5 (4)	C1—O2—Ce2^iii^	144.9 (3)
O2—C1—C2	118.9 (3)	C10—O3—Ce1	154.5 (3)
O1—C1—C2	118.6 (3)	C10—O3—Ce2	93.0 (2)
C1—C2—C3	115.1 (3)	Ce1—O3—Ce2	111.37 (9)
C1—C2—H2A	108.5	C10—O4—Ce2	95.8 (2)
C3—C2—H2A	108.5	C11—O5—Ce2	143.3 (3)
C1—C2—H2B	108.5	C11—O6—Ce1	130.5 (2)
C3—C2—H2B	108.5	C20—O7—Ce2^vii^	96.4 (2)
H2A—C2—H2B	107.5	C20—O8—Ce2^v^	133.5 (2)
C4—C3—C8	118.4 (4)	C20—O8—Ce2^vii^	92.4 (2)
C4—C3—C2	122.1 (3)	Ce2^v^—O8—Ce2^vii^	114.00 (10)
C8—C3—C2	119.5 (4)	C21—O9—Ce1	97.1 (2)
C5—C4—C3	120.4 (4)	C21—O10—Ce2	149.1 (2)
C5—C4—H4	119.8	C21—O10—Ce1	92.4 (2)
C3—C4—H4	119.8	Ce2—O10—Ce1	108.93 (9)
C6—C5—C4	119.8 (4)	C30—O11—Ce1^iv^	102.5 (2)
C6—C5—H5	120.1	C30—O12—Ce1^i^	156.6 (3)
C4—C5—H5	120.1	C30—O12—Ce1^iv^	88.1 (2)
C5—C6—C7	121.2 (4)	Ce1^i^—O12—Ce1^iv^	114.26 (9)
C5—C6—H6	119.4	Ce1—O13—H1W	120 (3)
C7—C6—H6	119.4	Ce1—O13—H2W	111 (4)
C6—C7—C8	118.4 (4)	H1W—O13—H2W	114 (3)
C6—C7—C9	121.5 (4)	Ce2—O14—H3W	109 (3)
C8—C7—C9	120.1 (4)	Ce2—O14—H4W	119 (4)
C7—C8—C3	121.7 (4)	H3W—O14—H4W	112 (3)
			
O2—C1—C2—C3	23.6 (5)	O3—C10—O4—Ce2	4.4 (4)
O1—C1—C2—C3	−156.0 (3)	C9—C10—O4—Ce2	−173.2 (4)
C1—C2—C3—C4	−66.9 (5)	O2^iv^—Ce2—O4—C10	94.5 (3)
C1—C2—C3—C8	112.7 (4)	O5—Ce2—O4—C10	−73.3 (2)
C8—C3—C4—C5	−1.1 (6)	O8^v^—Ce2—O4—C10	−124.9 (2)
C2—C3—C4—C5	178.5 (4)	O10—Ce2—O4—C10	−7.0 (3)
C3—C4—C5—C6	0.8 (7)	O14—Ce2—O4—C10	68.4 (2)
C4—C5—C6—C7	0.5 (7)	O7^vi^—Ce2—O4—C10	138.2 (2)
C5—C6—C7—C8	−1.5 (7)	O3—Ce2—O4—C10	−2.4 (2)
C5—C6—C7—C9	−179.4 (4)	O8^vi^—Ce2—O4—C10	−171.6 (2)
C6—C7—C8—C3	1.2 (6)	C20^vi^—Ce2—O4—C10	163.0 (3)
C9—C7—C8—C3	179.1 (4)	O6—C11—O5—Ce2	37.2 (7)
C4—C3—C8—C7	0.1 (6)	C12—C11—O5—Ce2	−143.5 (4)
C2—C3—C8—C7	−179.5 (4)	O2^iv^—Ce2—O5—C11	−99.8 (5)
C6—C7—C9—C10	−62.4 (6)	O8^v^—Ce2—O5—C11	−149.8 (5)
C8—C7—C9—C10	119.7 (5)	O10—Ce2—O5—C11	−56.8 (5)
C7—C9—C10—O4	−20.7 (7)	O4—Ce2—O5—C11	67.8 (5)
C7—C9—C10—O3	161.6 (4)	O14—Ce2—O5—C11	15.4 (5)
O5—C11—C12—C13	170.6 (4)	O7^vi^—Ce2—O5—C11	119.9 (4)
O6—C11—C12—C13	−10.1 (6)	O3—Ce2—O5—C11	16.2 (4)
C11—C12—C13—C14	−59.1 (6)	O8^vi^—Ce2—O5—C11	147.9 (5)
C11—C12—C13—C18	123.2 (4)	C20^vi^—Ce2—O5—C11	134.6 (4)
C18—C13—C14—C15	−0.3 (7)	O5—C11—O6—Ce1	−28.8 (6)
C12—C13—C14—C15	−178.1 (5)	C12—C11—O6—Ce1	152.0 (3)
C13—C14—C15—C16	1.1 (9)	O3—Ce1—O6—C11	−25.8 (3)
C14—C15—C16—C17	−1.8 (9)	O12^i^—Ce1—O6—C11	142.7 (4)
C15—C16—C17—C18	1.6 (8)	O1^ii^—Ce1—O6—C11	−24.1 (4)
C15—C16—C17—C19	−178.8 (5)	O11^iii^—Ce1—O6—C11	−101.8 (4)
C16—C17—C18—C13	−0.7 (6)	O13—Ce1—O6—C11	−179.6 (3)
C19—C17—C18—C13	179.7 (4)	O9—Ce1—O6—C11	79.8 (4)
C14—C13—C18—C17	0.1 (6)	O10—Ce1—O6—C11	46.0 (3)
C12—C13—C18—C17	177.9 (4)	O12^iii^—Ce1—O6—C11	−150.1 (4)
C16—C17—C19—C20	70.5 (6)	C30^iii^—Ce1—O6—C11	−125.2 (4)
C18—C17—C19—C20	−109.9 (5)	O8—C20—O7—Ce2^vii^	8.7 (4)
C17—C19—C20—O7	−8.7 (6)	C19—C20—O7—Ce2^vii^	−168.8 (3)
C17—C19—C20—O8	173.7 (4)	O7—C20—O8—Ce2^v^	118.5 (4)
O9—C21—C22—C23	3.2 (6)	C19—C20—O8—Ce2^v^	−63.9 (5)
O10—C21—C22—C23	−178.3 (3)	Ce2^vii^—C20—O8—Ce2^v^	126.9 (3)
C21—C22—C23—C24	65.8 (5)	O7—C20—O8—Ce2^vii^	−8.5 (4)
C21—C22—C23—C28	−114.4 (4)	C19—C20—O8—Ce2^vii^	169.1 (3)
C28—C23—C24—C25	0.7 (6)	O10—C21—O9—Ce1	−10.2 (4)
C22—C23—C24—C25	−179.5 (4)	C22—C21—O9—Ce1	168.2 (3)
C23—C24—C25—C26	0.2 (7)	O3—Ce1—O9—C21	40.0 (2)
C24—C25—C26—C27	−1.1 (7)	O12^i^—Ce1—O9—C21	−102.8 (2)
C25—C26—C27—C28	1.1 (6)	O1^ii^—Ce1—O9—C21	102.3 (2)
C25—C26—C27—C29	−176.6 (4)	O11^iii^—Ce1—O9—C21	143.4 (2)
C26—C27—C28—C23	−0.3 (6)	O6—Ce1—O9—C21	−40.1 (2)
C29—C27—C28—C23	177.4 (4)	O13—Ce1—O9—C21	175.9 (2)
C24—C23—C28—C27	−0.6 (6)	O10—Ce1—O9—C21	5.7 (2)
C22—C23—C28—C27	179.6 (4)	O12^iii^—Ce1—O9—C21	−142.6 (2)
C26—C27—C29—C30	80.0 (5)	C30^iii^—Ce1—O9—C21	−169.3 (2)
C28—C27—C29—C30	−97.7 (5)	O9—C21—O10—Ce2	−124.7 (4)
C27—C29—C30—O11	6.5 (6)	C22—C21—O10—Ce2	56.8 (6)
C27—C29—C30—O12	−172.7 (4)	O9—C21—O10—Ce1	9.9 (4)
O2—C1—O1—Ce1^ii^	174.8 (3)	C22—C21—O10—Ce1	−168.6 (3)
C2—C1—O1—Ce1^ii^	−5.6 (7)	O2^iv^—Ce2—O10—C21	−9.9 (5)
O1—C1—O2—Ce2^iii^	−39.5 (7)	O5—Ce2—O10—C21	−163.4 (5)
C2—C1—O2—Ce2^iii^	141.0 (4)	O8^v^—Ce2—O10—C21	−91.3 (5)
O4—C10—O3—Ce1	158.7 (4)	O4—Ce2—O10—C21	129.7 (5)
C9—C10—O3—Ce1	−23.6 (8)	O14—Ce2—O10—C21	62.0 (5)
O4—C10—O3—Ce2	−4.4 (4)	O7^vi^—Ce2—O10—C21	20.1 (6)
C9—C10—O3—Ce2	173.4 (4)	O3—Ce2—O10—C21	125.9 (5)
O12^i^—Ce1—O3—C10	−100.0 (6)	O8^vi^—Ce2—O10—C21	−88.0 (5)
O1^ii^—Ce1—O3—C10	100.2 (6)	C20^vi^—Ce2—O10—C21	−17.6 (7)
O11^iii^—Ce1—O3—C10	11.8 (6)	O2^iv^—Ce2—O10—Ce1	−141.20 (12)
O6—Ce1—O3—C10	−80.9 (6)	O5—Ce2—O10—Ce1	65.30 (10)
O13—Ce1—O3—C10	77.0 (6)	O8^v^—Ce2—O10—Ce1	137.37 (11)
O9—Ce1—O3—C10	165.4 (6)	O4—Ce2—O10—Ce1	−1.62 (14)
O10—Ce1—O3—C10	−167.3 (6)	O14—Ce2—O10—Ce1	−69.27 (11)
O12^iii^—Ce1—O3—C10	−12.3 (6)	O7^vi^—Ce2—O10—Ce1	−111.17 (16)
C30^iii^—Ce1—O3—C10	0.9 (6)	O3—Ce2—O10—Ce1	−5.37 (9)
O12^i^—Ce1—O3—Ce2	61.73 (18)	O8^vi^—Ce2—O10—Ce1	140.76 (15)
O1^ii^—Ce1—O3—Ce2	−98.04 (11)	C20^vi^—Ce2—O10—Ce1	−148.9 (3)
O11^iii^—Ce1—O3—Ce2	173.59 (13)	O3—Ce1—O10—C21	−151.6 (2)
O6—Ce1—O3—Ce2	80.89 (11)	O12^i^—Ce1—O10—C21	61.2 (2)
O13—Ce1—O3—Ce2	−121.29 (15)	O1^ii^—Ce1—O10—C21	−80.3 (2)
O9—Ce1—O3—Ce2	−32.85 (13)	O11^iii^—Ce1—O10—C21	−153.0 (2)
O10—Ce1—O3—Ce2	−5.56 (9)	O6—Ce1—O10—C21	134.7 (2)
O12^iii^—Ce1—O3—Ce2	149.44 (9)	O13—Ce1—O10—C21	−16.5 (2)
C30^iii^—Ce1—O3—Ce2	162.69 (11)	O9—Ce1—O10—C21	−5.4 (2)
O2^iv^—Ce2—O3—C10	−128.2 (2)	O12^iii^—Ce1—O10—C21	92.7 (3)
O5—Ce2—O3—C10	94.8 (2)	C30^iii^—Ce1—O10—C21	165.4 (3)
O8^v^—Ce2—O3—C10	114.8 (2)	O3—Ce1—O10—Ce2	5.70 (9)
O10—Ce2—O3—C10	178.1 (2)	O12^i^—Ce1—O10—Ce2	−141.55 (11)
O4—Ce2—O3—C10	2.4 (2)	O1^ii^—Ce1—O10—Ce2	76.92 (11)
O14—Ce2—O3—C10	−85.8 (2)	O11^iii^—Ce1—O10—Ce2	4.2 (2)
O7^vi^—Ce2—O3—C10	−41.1 (2)	O6—Ce1—O10—Ce2	−68.03 (11)
O8^vi^—Ce2—O3—C10	16.5 (3)	O13—Ce1—O10—Ce2	140.74 (11)
C20^vi^—Ce2—O3—C10	−15.0 (3)	O9—Ce1—O10—Ce2	151.85 (16)
O2^iv^—Ce2—O3—Ce1	59.62 (14)	O12^iii^—Ce1—O10—Ce2	−110.06 (18)
O5—Ce2—O3—Ce1	−77.39 (11)	C30^iii^—Ce1—O10—Ce2	−37.3 (4)
O8^v^—Ce2—O3—Ce1	−57.48 (16)	O12—C30—O11—Ce1^iv^	1.3 (4)
O10—Ce2—O3—Ce1	5.85 (9)	C29—C30—O11—Ce1^iv^	−177.9 (3)
O4—Ce2—O3—Ce1	−169.87 (16)	O11—C30—O12—Ce1^i^	162.1 (5)
O14—Ce2—O3—Ce1	101.99 (12)	C29—C30—O12—Ce1^i^	−18.7 (9)
O7^vi^—Ce2—O3—Ce1	146.68 (10)	Ce1^iv^—C30—O12—Ce1^i^	163.2 (7)
O8^vi^—Ce2—O3—Ce1	−155.72 (8)	O11—C30—O12—Ce1^iv^	−1.1 (4)
C20^vi^—Ce2—O3—Ce1	172.81 (11)	C29—C30—O12—Ce1^iv^	178.0 (4)

Symmetry codes: (i) −*x*+2, −*y*+3, −*z*+2; (ii) −*x*+2, −*y*+1, −*z*+1; (iii) *x*, *y*−1, *z*; (iv) *x*, *y*+1, *z*; (v) −*x*+1, −*y*+2, −*z*+2; (vi) *x*, *y*, *z*−1; (vii) *x*, *y*, *z*+1.

### Hydrogen-bond geometry (Å, °)

**Table:**

*D*—H···*A*	*D*—H	H···*A*	*D*···*A*	*D*—H···*A*
O13—H1W···O6^viii^	0.86 (1)	2.03 (2)	2.860 (4)	161 (4)
O14—H4W···O1^ii^	0.87 (4)	2.23 (2)	3.051 (4)	159 (5)
O14—H3W···O9^ix^	0.86 (4)	2.62 (4)	3.180 (4)	123 (4)
O14—H3W···O1^iv^	0.86 (4)	2.07 (5)	2.873 (4)	155 (5)
O13—H2W···O9	0.86 (5)	2.81 (7)	3.037 (4)	97 (5)
O13—H2W···O1^ii^	0.86 (5)	2.75 (5)	2.898 (4)	91 (3)

Symmetry codes: (viii) −*x*+2, −*y*+2, −*z*+2; (ii) −*x*+2, −*y*+1, −*z*+1; (ix) −*x*+2, −*y*+2, −*z*+1; (iv) *x*, *y*+1, *z*.
